# Advanced Strategies to Tailor the Nucleation and Crystal Growth in Hybrid Halide Perovskite Thin Films

**DOI:** 10.3389/fchem.2022.842924

**Published:** 2022-04-13

**Authors:** Jitendra Kumar, Priya Srivastava, Monojit Bag

**Affiliations:** ^1^ Advanced Research in Electrochemical Impedance Spectroscopy, Department of Physics, Indian Institute of Technology Roorkee, Roorkee, India; ^2^ Centre of Nanotechnology, Indian Institute of Technology Roorkee, Roorkee, India

**Keywords:** perovskite, nucleation and growth, nanocrystal, anti-solvent (or dilution) crystallization, thin film

## Abstract

Remarkable improvement in the perovskite solar cell efficiency from 3.8% in 2009 to 25.5% today has not been a cakewalk. The credit goes to various device fabrication and designing techniques employed by the researchers worldwide. Even after tremendous research in the field, phenomena such as ion migration, phase segregation, and spectral instability are not clearly understood to date. One of the widely used techniques for the mitigation of ion migration is to reduce the defect density by fabricating the high-quality perovskite thin films. Therefore, understanding and controlling the perovskite crystallization and growth have become inevitably crucial. Some of the latest methods attracting attention are controlling perovskite film morphology by modulating the coating substrate temperature, antisolvent treatment, and solvent engineering. Here, the latest techniques of morphology optimization are discussed, focusing on the process of nucleation and growth. It can be noted that during the process of nucleation, the supersaturation stage can be induced faster by modifying the chemical potential of the system. The tailoring of Gibbs free energy and, hence, the chemical potential using the highly utilized techniques is summarized in this minireview. The thermodynamics of the crystal growth, design, and orientation by changing several parameters is highlighted.

## Introduction

Hybrid halide perovskites (HHPs) have appeared in a supporting role as visible light sensitizers (Kojima et al., 2009) in the solar cell industry and have suddenly stolen the spotlight by emerging as an active material for high-efficiency solar cells (Heo et al., 2016; Zhao Y. et al., 2018, Zhao et al., 2018 B.). Perovskite solar cells (PSCs) have reached the efficiency of 25.5%, (NREL) which is equivalent to that of crystalline Si solar cells from merely 3.8% in 2009 (Kojima et al., 2009). This extraordinary success of these materials comes owing to their astonishing optoelectronic properties such as long charge carrier diffusion length (Zhang et al., 2017), large carrier lifetime (Wehrenfennig et al., 2014), high absorption coefficient (Chen et al., 2015), ambipolar charge transport (Srivastava and Bag, 2020), and tunable band gap (Atourki et al., 2016). However, such escalation in the efficiency of PSCs, in almost a decade, has not been a cakewalk. The credit goes to the researchers working around the globe employing various device fabrication and designing techniques. These techniques include optimizing the perovskite thin film morphology (Li et al., 2018), incorporating transport layers and interfacial engineering for better charge extraction and band alignment in device architecture (Liu et al., 2016; Srivastava et al., 2021b), band gap tuning of the perovskite active layer in order to match the absorption of the active layer with the AM 1.5 solar spectrum (Atourki et al., 2016), defect passivation (Zheng et al., 2017), and trap elimination (Yu et al., 2018). Although there have been a huge scientific interest and attention in this field, scientists still need to reach a consensus on the exact reason behind such a phenomenal success of these materials even in the amorphous phase. Perovskites combine the properties of inorganic materials such as high photoluminescence quantum yield (Lin et al., 2018), long carrier diffusion lengths (Shi et al., 2015), high color purity to that with the properties of organic materials such as solution processibility (Rodríguez-Gutiérrez et al., 2018; Wang P. et al., 2019) at low temperature, and high production yield. Given that the HHP materials can be stabilized (Bag et al., 2015; Nie et al., 2016), these materials have the potential to disrupt the mature silicon solar cell market. Even though the efficiencies of the HHP-based solar cells and light-emitting diodes are reaching their peak values, there are still several phenomena such as ion migration (Zhang T. et al., 2019; Srivastava et al., 2021a), phase segregation (Samu et al., 2019), and spectral instability (Mondal et al., 2019), which are not fully understood to date.

Due to the interdependence of material properties, they are entangled in a greatly complex way. Major complexity arises due to ion migration which is the main reason for the degradation in perovskites. One of the highly used strategies to minimize ion migration is the reducing defect density in the crystal structure by fabricating the high-quality perovskite thin films. In this review, several reports focusing on understanding and controlling the crystallization process to achieve some best quality perovskite films are discussed. The perovskite film morphology is a key factor in deciding the carrier lifetime, the carrier diffusion lengths, and ultimately the device performance (Ko et al., 2015). For the high-performance perovskite solar cells, large grains with interconnected grain boundaries are needed, while for the high-efficiency light-emitting diodes, small grains with pinhole-free film morphology are essential. Therefore, it is crucial to understand the crystal growth and control in accordance with the application. Good perovskite film morphology does not always refer to a smooth, pinhole-free, and compact perovskite film but is also expected to have favorable interfacial electrical properties. Using the antisolvent treatment and controlling the dripping delay, Kumar et al. demonstrated that film morphology can be tuned by tuning the route of the crystal growth, and thus, both the smooth and the textured perovskite film can be designed accordingly (Kumar R. et al., 2020). These textured films show better charge injection/extraction properties at the perovskite/electron transport layer interface in the perovskite light-emitting diode as compared to the archetypical smooth films.

In the last decade, a vast amount of research to maximize the efficiency of the HHP-based devices is based on the optimization of the hybrid perovskite crystal design, size, and growth orientation. A plethora of strategies and methods have been used to obtain uniform, pinhole-free, full coverage, and smooth perovskite layers. These methods involve changing the perovskite precursor solution concentration and temperature, optimizing the coating parameters such as the spin speed and time, tuning the annealing temperature of thin films. In addition to these methods, new techniques such as composition (Kang and Park, 2019) and thermal engineering (Srivastava et al., 2018), additive engineering in the perovskite precursor solution (Tavakoli et al., 2019; Giuliano et al., 2021; Zhang et al., 2021), air-assisted drying (Ding et al., 2019), humidity tuning (Gangishetty et al., 2016), vapor-assisted annealing (Sheng et al., 2015; Shi et al., 2015; Karlsson et al., 2021), and surface passivation layer capping (Chen P. et al., 2018; Tavakoli et al., 2019; He et al., 2020; Yu et al., 2021) are also employed. To gain a deeper insight into this, we need to understand film optimization through the process of the crystal growth and design. Recently, three techniques which have gained attention and provided advanced mechanisms to optimize the film morphology by controlling the crystallization and growth mechanism are regulating the substrate temperature, antisolvent treatment, and solvent engineering. In this review, we focused on these latest advanced techniques for the controlled perovskite crystal growth and design. The basic model of nucleation and growth of the perovskite crystals during thin film fabrication is described. Latest articles and research on this topic will be reviewed and analyzed to summarize a generalized nucleation and growth model.

## Nucleation and Growth of Perovskite Crystals

In any solution processable technique, such as spin coating, crystal growth can be considered as a two-step process; the first step is the nucleus formation (nucleation), and the second step is the growth of nuclei into larger crystals. The nucleation of crystals can be of two types: homogeneous and heterogeneous nucleation. Homogeneous nucleation occurs spontaneously without any external assistance within the solution. However, heterogeneous nucleation requires an external site to occur (e.g., colloidal particles in the solution and substrate surfaces). Both of these types of nucleation can occur in the solution on the colloidal particle sites. The produced nuclei can assemble into bulk clusters and grow into exaggerated film morphology when dropped on a substrate, leading to incompact films with incomplete coverage and a large number of defects. Therefore, nucleation on the substrate is the ideal approach for a high-quality perovskite film.

Nucleus formation on the substrate is initiated when the solution is supersaturated and the concentration of the precursor solution is sufficiently higher than its solubility. The solution in the supersaturated state is prerequisite to yield pure perovskite phases. This supersaturation stage can be achieved sooner by preconditioning the precursor or the coating substrate. One of the commonly used techniques to assist this process is the temperature treatment. The input of thermal energy facilitates a faster nucleation process, leading to uniform perovskite thin films. In addition, the intermediate phases or the perovskite precursor solvate (solvent–solute interaction) significantly influences the final perovskite film formation. These always occur in the thin film during the processing methods such as antisolvent treatment or solvent engineering.

Nucleation and growth are best understood in terms of chemical potential (µ), which in the simplest term is the energy released or absorbed by the addition (removal) of a particle into (from) the system. At a constant pressure and constant temperature, which is the archetypical lab conditions for solution processing, the chemical potential (µ) can be calculated from a thermodynamic potential called Gibbs free energy (G).

$$\begin{array}{c} \mu ={\left(\frac{\partial G}{\partial Ni}\right)}_{T,P,N_{i\neq j}} \end{array},$$ (1)

The process of nucleation and growth can be tuned by regulating the chemical potential of the thermodynamic system. There are several thin film processing methods by which the chemical potential of the nucleic site can be altered. Few of the latest techniques being followed these days include the variation in the substrate or solvent temperature and variation in the rate of solvent extraction at the time of spin coating or by changing the vapor pressure of the ink, which have to be used in order to fabricate perovskite films. Here, we focused on how the crystallization kinetics is modulated and the crystals are designed to yield a high-quality perovskite thin film by changing the substrate temperature before film fabrication, antisolvent treatment of the film during spin coating, and solvent engineering of the precursor solution.

## Crystallization Control by Substrate Temperature Treatment

**FIGURE 1:**
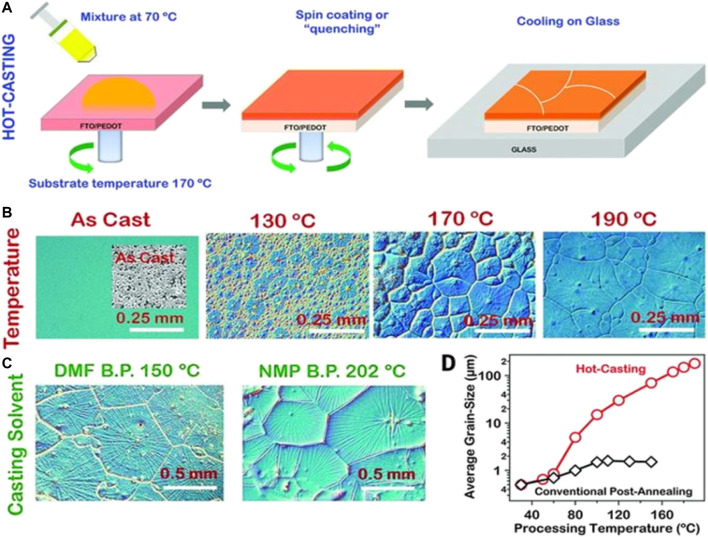
Processing scheme for the perovskite thin film using hot-casting methods and observations for a large-area millimeter-scale crystal grain formation for a perovskite (PbCH_3_NH_3_I_3–x_Cl_x_)–based thin film. **(A)** Hot-casting scheme for a large-area crystal growth [ITO, indium tin oxide; FTO, fluorine-doped tin oxide; PEDOT:PSS, poly (3,4-ethylenedioxythiophene) polystyrene sulfonate]. **(B)** Optical micrographs illustrating the grain formation as a function of the substrate temperature with the casting solution maintained at 70°C. **(C)** Large-area grain formation using the casting solvents with high boiling points. **(D)** Comparison of the grain size as a function of the processing temperature obtained for the hot-casting and conventional post-annealing methods. Reproduced with permission (Nie et al., 2015). Copyright 2015, American Association for the Advancement of Science.

We have already discussed earlier that the formation of perovskite crystals is a two-step process: nucleation and growth. In addition, during the complete process, the temperature plays a very significant role. The chemical potential (μ) and, hence, Gibbs free energy (G) of the system can be significantly tuned by modulating the temperature of the process. The temperature of the system can be changed either during nucleation or during growth. The fabrication of the perovskite thin films using spin coating involves mainly two steps. The first is spin coating the precursor solution onto the substrate (nucleation) and annealing the coated substrate at an optimized temperature (growth). In this article, we focused on how the morphology of the perovskite thin film can be tuned by playing with the process of nucleation of the perovskite crystals. The process of nucleation can be controlled either by changing the temperature of the precursor solution before spin coating or preheating the substrate. The technique of preheating the substrate before coating the perovskite layer has been explored recently. It is one of the latest advanced strategies used to modulate the mechanism of crystal growth and, hence, the crystal design. There have been few studies on the effect of substrate temperature on the morphology of the perovskite film. Among some of the earlier studies is a report by Tidhar et al. (2014) in which the SEM images of methylammonium lead bromide (MAPbBr_3_) films coated on substrates with different temperatures were examined. It was noted that preheating the substrate affects the degree of surface-induced nucleation. With the increase in temperature, the number of nucleation events increased, leading to a larger number of smaller crystals and hence better coverage. In another study, Zheng et al. demonstrated the thermally induced Volmer–Weber growth mechanism during the hot casting of methylammonium lead iodide (MAPbI_3_) perovskite films (Zheng et al., 2015). In this report, the partially coated perovskite thin film transforms from the branch-shaped morphology to island-shaped and then finally evolves to a full coverage film with densely packed islands with the increase in the casting temperature. It is suggested that the thermally directed thermodynamics of the crystalline perovskite film formation decides the morphology and film growth mode. Later, Nie et al. demonstrated a solution-based hot-casting technique to grow pinhole-free and continuous perovskite thin films with the crystalline grains of a millimeter scale, resulting in hysteresis-free solar cells with efficiencies approaching 18% (Nie et al., 2015). Figure 1 shows the schematic representation of the hot-casting technique used, which involves the casting of a hot (∼70°C) mixture of lead iodide (PbI_2_) and methylammonium chloride (MACl) solution onto the substrate maintained at temperature of up to 180°C followed by a subsequent spin coating of the precursor. They have attributed that the excess solvent present on the substrate at a temperature above the crystallization temperature for the formation of the perovskite phase allows the prolonged growth of the perovskite crystals and hence yielding large crystalline grains.

**FIGURE 2:**
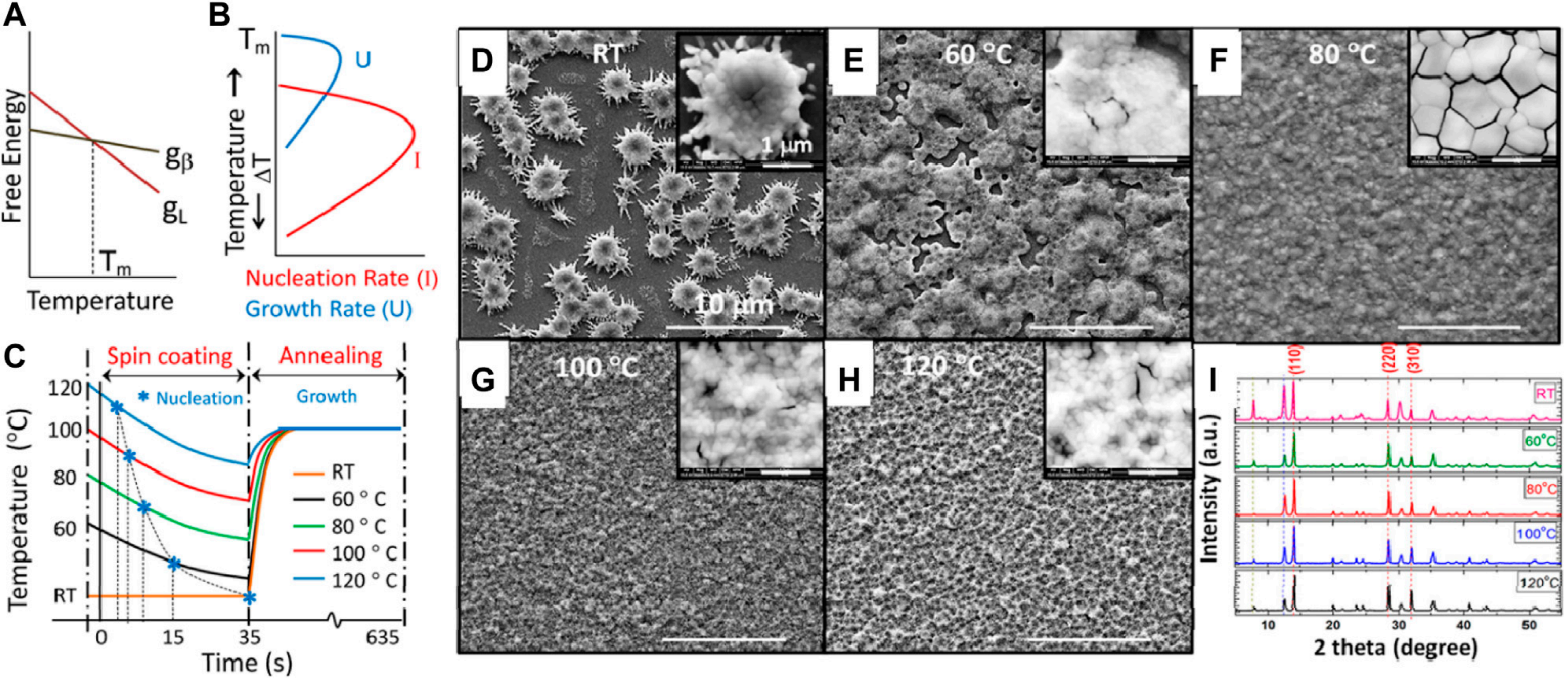
**(A)** Schematic variation of the free energy of a material in a liquid (g_L_) and crystalline form (g_β_) as a function of temperature. **(B)** Temperature dependence of nucleation rate (I) and growth rate (U). Adopted with permission from Raghavan, 2011. Copyright 2011 Prentice-Hall India. **(C)** Expected temperature variation (not true scale) of the substrates during spin coating and annealing (assuming Newtonian cooling during spin coating and at thermal equilibrium during annealing). Blue asterisks indicate the nucleation points where yellow-colored films turn dark-colored. **(D–H)** FESEM images of the perovskite film fabricated at different substrate temperatures. The scale is 10 μm. Inset: zoomed FESEM images of each film with the scale bar of 1 μm. **(I)** XRD of the perovskite film fabricated at different substrate temperatures. Peaks indicated by red dotted lines (∼14°, ∼28.5°, and ∼32°) correspond to the characteristic MAPbI3 peak. Peaks indicated by the blue dotted line (∼12.6°) correspond to the lead iodide peak, and black dotted line (∼7.9°) corresponds to the lead acetate peak. Reproduced with permission (Srivastava et al., 2018). Copyright 2018, American Chemical Society.

In 2018, Srivastava et al. described the underlying theory of crystallization thermodynamics altered by the preheating temperature which leads to a significant change in the film morphology (Srivastava et al., 2018). In a single-step deposition process of the perovskite film, when the precursor is spin coated on the substrate, phase transformation takes place from the liquid (L) to the crystalline (β) phase. The variation of free energy for the two phases as a function of temperature is shown in Figure 2A. The value of ΔG is zero at the equilibrium melting point T_m_. Above T_m_, ΔG > 0 is not favorable for spontaneous transformation, whereas below T_m_, ΔG < 0 favors spontaneous phase transformation. In this study, the perovskite film was coated on the substrates preheated to five temperatures below T_m_, (T_m_ for MAPbI_3_ = 120°C).

Once the condition for spontaneous phase transformation is satisfied, homogeneous nucleation with the nucleating particle of a critical radius is preferred for the uniform and full coverage films. The rate of nucleation (*I*) is given by the product of the number of critical sized particles (N*) and the frequency at which they become supercritical, i.e., the frequency at which they cross the interface to join the product phase (*ν′*), as given by the equation (Raghavan, 2011)

$$\begin{array}{c} I=N^{*}\nu ,=N_{t}s^{*}\nu exp\left(-\frac{\Delta f^{*}+\Delta H_{d}}{RT}\right) \end{array},$$ (2)

where $N_{t}$ is the number density of atoms in the parent phase, $s^{*}$ is the number of atoms in the parent phase facing the critical sized particles, ν is the lattice vibration frequency, $\Delta f^{*}$ is the free energy change for the formation of a spherical particle with critical radius *r* ^*^, $\Delta H_{d}$ is the activation energy for the diffusion across the interface, *R* is the gas constant, and *T* is the temperature of the transformation. The temperature dependance of the nucleation rate (*I*) according to this equation is shown in Figure 2A. It shows that the nucleation rate is maximum at temperature just below the T_m_, which agrees with the observed results. It was found that the perovskite film coated on the substrate with a preheating temperature of 100°C has an optimum morphology for the high open-circuit voltage and uniform charge and ion diffusion across the interface. Moreover, the dependence of the growth rate (U) with the temperature reveals that the growth of the nucleated particles is maximum at a temperature just below the maximum nucleation temperature. Hence, in this study, the films were annealed at the temperature of 100°C for 10 min. This study suggests that the mechanism of fast nucleation followed by delayed growth is the best method to obtain high-quality perovskite films (Figure 2). A similar observation was also noted by Li et al. in their study of effect of substrate temperature on the microstructure of PbI_2_ films (Li et al., 2020). They reported that increasing the processing substrate temperature eliminates the larger voids in the films leading to uniform films with distributed surface pores. This is attributed to the enhanced solvent evaporation rates, which reduces solvent dewetting at the substrate and increases the nucleation rates of PbI_2_ crystals to yield more uniform and homogeneous films.

The temperature-assisted nucleation of the perovskite crystals was also utilized for the fabrication of large-area perovskite films using a solution shearing technique by Shin and group (Kim et al., 2018). They reported that with the increase in the substrate temperature, the evaporation rate and, hence, supersaturation increases drastically, and this allows the system to bypass the intermediate solvent states leading to the direct formation of granules. A similar observation was also made by Zhang H. et al. (2019). They reported that the color of the freshly prepared perovskite film changes from reddish brown to charcoal with an increase in temperature. The exhibited red color is considered as an intermediated phase, and hence, the portion of the intermediate phase drops as the substrate temperature increases. They claimed that the fresh perovskite films coated at a low substrate temperature have a pure intermediate phase with a uniform dendritic structure which transforms into smooth cubic phase films after annealing. However, in the films coated at higher substrate temperature, the δ phase begins to appear along with the intermediate phase. As a result, the morphology of the fresh perovskite films changes to the coexisting dendritic and island-like structure. After annealing, the original island morphology still remains and leads to a rough surface.

**FIGURE 3:**
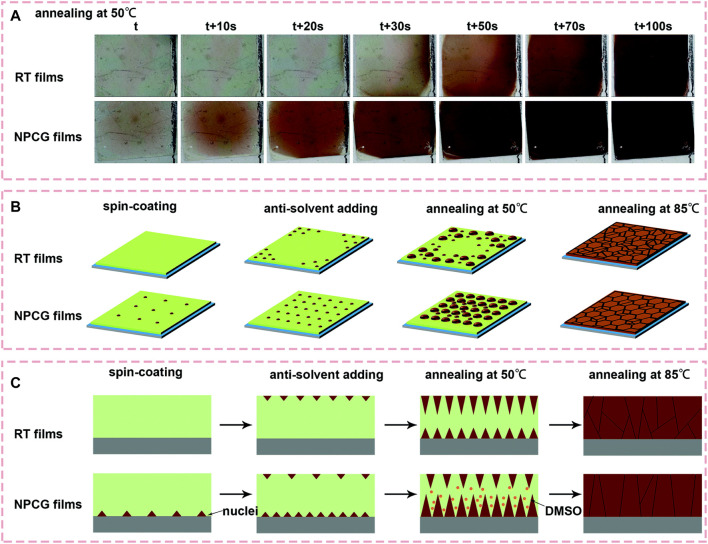
**(A)** Photos recorded during thermal annealing of the perovskite films using NPCG@0 and RT methods at a temperature of 50°C. Illustration of the nucleation and growth of the grains for the films fabricated using NPCG and RT methods at each stage, **(B)** top view and **(C)** cross-sectional view. Reproduced with permission (Kang and Park, 2019). Copyright 2019, The Royal Society of Chemistry.

In contrast to the fabricating perovskite thin films on the preheated substrates, Wang et al. developed a new method of spin coating the perovskite films on frozen substrates to control the position of the nuclei and the crystal growth orientation simultaneously (Wang G. et al., 2019). They introduced a new method called the nuclei position-control and growth-guidance (NPCG) method to obtain the high-quality perovskite films (Figure 3). According to their study, when the precursor is added to the cold substrate, nuclei are formed at the substrate side due to the lower solubility of the perovskite in the precursor at low temperature, and more dimethyl sulfoxide (DMSO) solvent is trapped in the film due to the slower evaporation rate than that at Room Temperature (RT). These uniformly distributed perovskite nuclei at the substrate side guide the crystal growth with the help of a residual solvent in the film (Figures 3B,C). During the process of thermal annealing, the grain boundaries are dissolved, and large grains connecting the bottom substrate to the top surface of the film are formed. Hence, high-quality perovskite films with crystals oriented perpendicular to the substrate with less cracked grain boundaries are obtained. Recently, these techniques of controlling the crystal growth and orientation have also been used for fabricating the perovskite films by the vacuum co-deposition method. Snaith et al., demonstrated the accurate control of the crystallite size in MAPbI_3_ thin films by modulating the substrate temperature during the vacuum co-deposition of the inorganic (PbI_2_) and organic methylammonium iodide (MAI) precursors (Lohmann et al., 2020). They found that the films co-deposited on the colder substrate (−2°C) exhibited larger micrometer-sized crystals, while the films fabricated on the substrate at RT (23°C) only produced grains of 100 nm extent. They reported that the substrate temperature directly affects the adsorption rate of MAI and thus impacts the crystal formation kinetics. In another study by Marcel et al., the impact of the substrate temperature on the co-evaporated p-i-n perovskite solar cell efficiency is investigated (Roß et al., 2020).

They analyzed the evaporation of pure precursor materials MAI and PbI_2_ and reported that the adhesion of MAI is significantly decreased at the elevated substrate temperature, while it remains almost unaltered for PbI_2_. This substrate temperature-dependent adhesion behavior of MAI highly influences the co-evaporation process and directly affects the perovskite composition in the film. They reported that the optimal substrate temperature window for the perovskite deposition is close to the room temperature. They demonstrated that at high temperature, even very high MAI rates cannot incorporate enough MAI for precise stoichiometry. While at low temperatures (<−25°C), the conversion of MAI with PbI_2_ is inhibited, leading to the formation of the unreacted amorphous film. Therefore, tailoring of the optimum substrate temperature for both spin coating (Ko et al., 2015; Chen J. et al., 2018) and vacuum co-evaporation(Parveen et al., 2020; Ghosh and Giri, 2021) is important to obtain the high-quality hybrid perovskite thin films with low defect density and required grain size.

## Crystallization Control by Antisolvent Treatment

Crystallization in the perovskite films can be controlled by the fast extraction of solvent with the help of an antisolvent. A fluid (liquid, vapor, or supercritical gas) can work as an antisolvent, given that it is miscible with the solvent of the perovskite ink; at the same time, perovskite precursors are expected to show poor miscibility with the antisolvent. The antisolvent treatment method helps the perovskite ink to reach the supersaturation state and form the nucleus sites. Formation of nuclei is governed by the variation in Gibbs free energy (G). For homogeneous nucleation, change in the Gibbs free energy is given by the following equation (Kumar et al., 2020a)

$$\begin{array}{c} \Delta G=\Delta G_{S}+\Delta G_{V}=4\pi r^{2}\gamma +\frac{4}{3}\pi r^{3}\Delta G_{V\;} \end{array},$$ (3)

where γ is the interfacial energy or surface energy between the supersaturated solution and crystalline surface, and ∆G_v_ is the bulk free energy per unit volume. From here, the critical free energy and critical nuclei radius can be estimated as (Lee et al., 2018)

$$\begin{array}{c} \Delta G^{*}=\frac{16\Pi \gamma }{3{\left(\Delta G_{v}\right)}^{2}};\;\;r^{*}=\frac{2\gamma }{\Delta G_{V}} \end{array},$$ (4)

To achieve the system with stable nuclei for further growth, the critical Gibbs free energy and critical radius of the nuclei need to be reduced. This requires a decrease in the surface energy between the supersaturated solution and the crystalline surface. The addition of additives such as benzylamine (PMA) in a/an solvent/antisolvent is known to decrease the surface energy (γ) and help in achieving uniform, compact, and small grain-sized perovskite films suitable for the high-performance light-emitting diodes (LEDs) (Lee et al., 2018).

**FIGURE 4:**
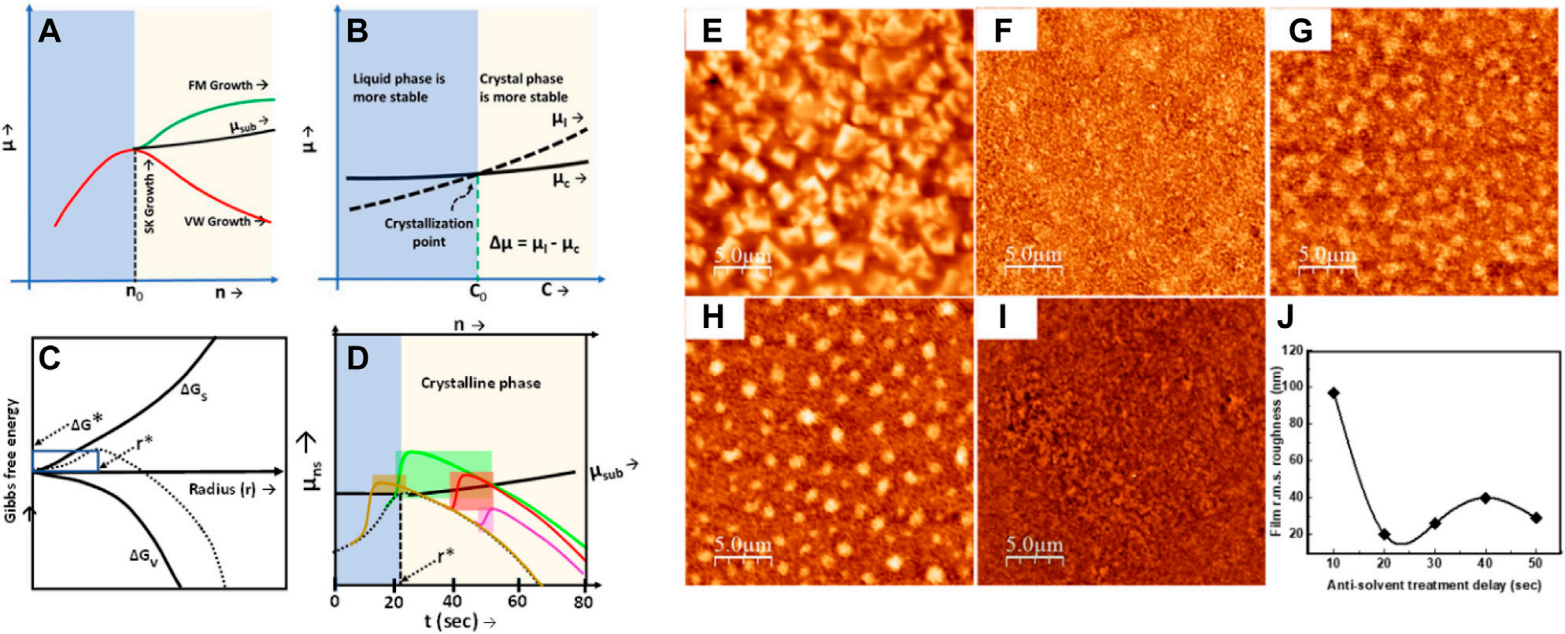
Schematic diagram of controlling the chemical potential with the help of antisolvent treatment during the crystallization of perovskite films. **(A)** Change in the chemical potential as a function of the adsorbed particles/molecules **(B)** Change in the chemical potential from a liquid phase to crystalline phase **(C)** Variation of Gibbs free energy as a function of particle radius and **(D)** Proposed variation of the chemical potential during the antisolvent dripping. Dotted line represents chemical energy variation without antisolvent dripping, while solid lines represent the chemical energy variation at different antisolvent dripping delay time, **(E–I)** film morphology obtained by dripping the antisolvent at 10, 20, 20, 40, and 50 s of time delay; **(J)** shows the variation in film roughness in perovskite films prepared at different antisolvent dripping delays. Reproduced with permission (Kumar et al., 2020). Copyright 2020, The Royal Society of Chemistry.

**FIGURE 5:**
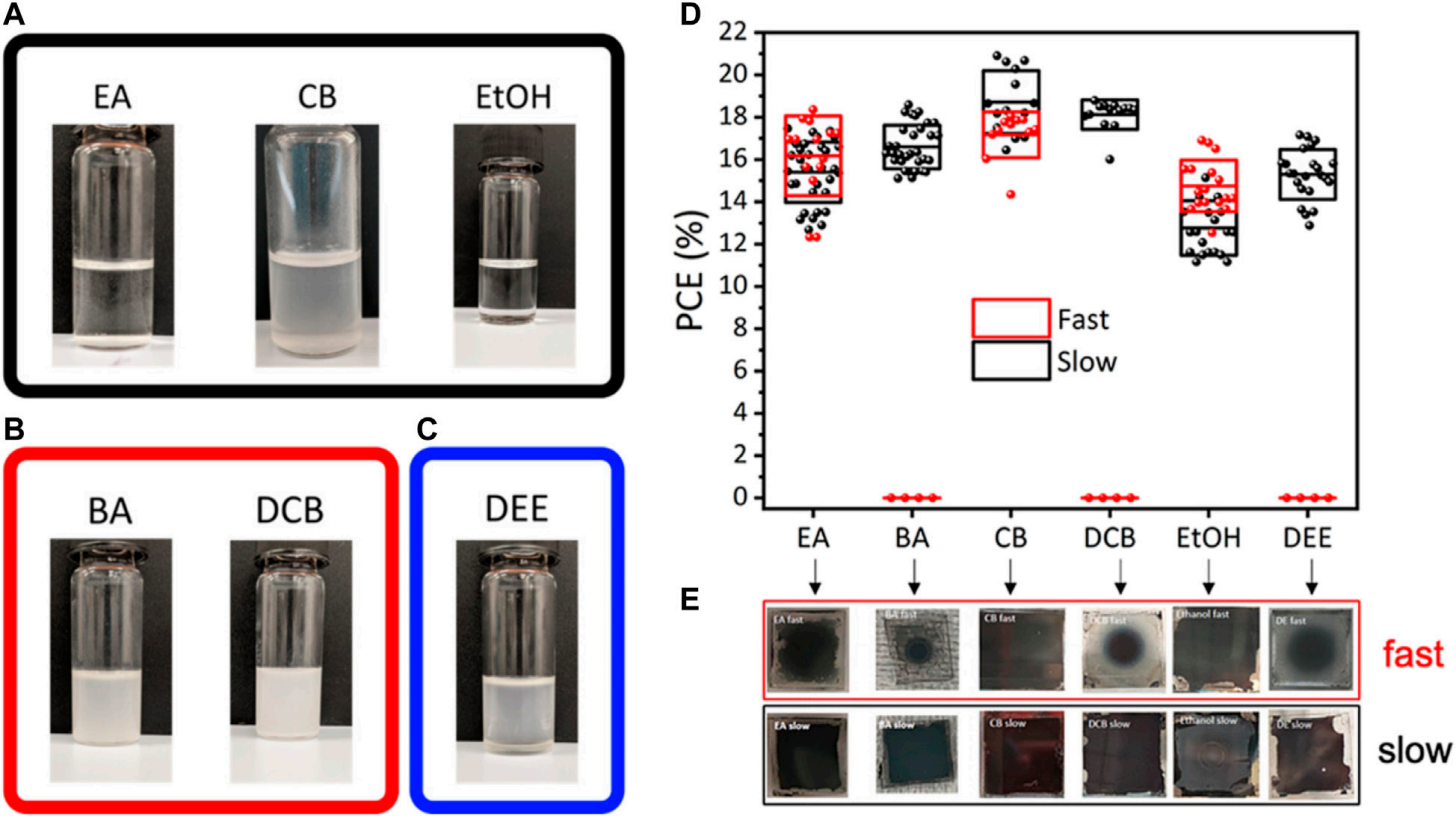
Precipitation tests using the six antisolvents studied in this work. 1 m solutions of CsI + FAI in DMF: DMSO, to which the antisolvent was added and immediately photographed. For **(A)** ethyl acetate, chlorobenzene, and ethanol, the solution remains clear, whereas for **(B)** butyl acetate and 1,2-dichlorobenzene, a turbid solution forms instead. **(C)** DEE forms a mostly clear solution; however, a liquid-phase separation is observed, **(D)** displays the effect of the antisolvent application rate on PCE of the perovskite solar cells, **(E)** shows the photographic image of the perovskite films with various combinations of antisolvents and antisolvent application rates. Reproduced with permission (An et al., 2021). Copyright 2021, Wiley.

Maxima in the Gibbs free energy can be considered as the starting point of phase transformation from a liquid to solid phase. Therefore, G_max_ corresponds to the equalization of chemical potentials of liquid and nucleus sites. Now, the role of the antisolvent treatment is to increase the chemical potential of the nucleus site and cause a positive gradient in the µ-n curve, which is the characteristic signature of Frank–van der Merwe (FM) growth. Thus, by tuning between the Volmer–Weber (VW) and FM growth, we controlled the crystallization dynamics to achieve the required film morphology. The volume of the antisolvent, dripping delay in antisolvent treatment, and the rate of pouring of the antisolvent can tune the perovskite film morphology by controlling the chemical potential of the nucleus sites. Kumar et el. used antisolvent dripping delay to achieve textured film morphology (Kumar et al., 2020). They found that the textured films take the Stranski–Krastanov (SK) growth kinetics to present micro islands on top of the smooth film. SK growth in the solution processable techniques is the combination of a highly stable Volmer–Weber (VW) growth mode and poorly stable Frank–van der Merwe (FM) growth mode. VW growth is characterized by the negative slope of µ_ns,_ which indicates a strong adatom–adatom interaction, whereas a positive differential of µ_ns_ implies a strong adatom–surface interaction, and it is responsible for the FM growth mode, as shown in Figure 4A. Therefore, SK growth can be seen as a positive derivative of the µ–n curve followed by a negative differential of µ afterward (Figure 4D, highlighted regions). The perovskite film was found to be decorated by the formation of micro islands with the base diameter in the range of 1–2 µm in size. The textured perovskite film shows improved photoluminescence, with an improved charge injection, and light outcoupling. The antisolvent dripping delay method can be utilized to prepare both the textured and smooth films by optimizing the dripping delay in an antisolvent treatment (Kumar et al., 2020). Vaynzof et al. have demonstrated the effect of the antisolvent application rate on the photovoltaic performance of the perovskite solar cells. They utilized six different antisolvents—ethyl acetate (EA), butyl acetate (BA), diethyl ether (DEE), ethanol (EtOH), chlorobenzene (CB), and 1,2-dichlorobenzene (DCB)—to test the effect of the application rate of an antisolvent. They found that the slow application (low rate of antisolvent dripping) of the antisolvent results in high-quality uniform films, leading to device efficiencies reaching more than 20%, whereas the fast application of the antisolvent results in incomplete coverage films and no functional perovskite solar cells when BA or DCB or DEE was utilized. On the other hand, EA, CB, or EtOH only show a slight decrease in the photovoltaic performance (An et al., 2021). In the precipitation test of A site molecule in a mixture of solvent and antisolvent mixtures, the antisolvents which resulted in only a slight decrease in the PV performance formed a clear solution with the prominent white precipitates at the bottom of the vial. However, those antisolvents that caused a huge drop in the PV performance and resulted in nonfunctional devices resulted in a cloudy, turbid suspension. Although DEE formed a transparent solution, the phase separation between dimethylformamide: dimethyl sulfoxide (DMF: DMSO) and DEE is observed, which indicates the poor miscibility of the antisolvent with the solvents for the perovskite ink (Figure 5C).

Recently, Jeong et al. achieved 25.6% power conversion efficiency in the perovskite solar cell using antisolvent treatment along with pseudo-halide anion engineering. They utilized diethyl as the antisolvent to the perovskite precursors dissolved in a mixture of DMF and DMSO (4:1) (Jeong et al., 2021). Although, the antisolvent treatment has resulted in the best performing devices, the commonly used antisolvents such as toluene, chlorobenzene (CB), and dichloromethane (DCM) are found to contaminate the drinking water due to their high toxicity level. Therefore, to scale up the production of the perovskite-based devices, we need to look forward to the eco-friendly antisolvents such as methoxybenzene (PhOMe) and Tert-butyl alcohol (tBuOH). Zhang et al. compared the performance of CB-treated and PhOMe-treated perovskite solar cells. They found that the perovskite solar cells prepared by PhOMe antisolvent treatment reached 19.42% PCE which is even better than CB processed PSCs (19.02%). They found that PhOMe treatment results in a larger grain size with reduced grain boundaries and defect density as compared to the CB-treated PSCs (Zhang et al., 2018). Similarly, Kim et al. used tBuOH as an alternative to the highly toxic antisolvents in a fully roll-to-roll processed PSCs (all layers except the electrodes) and achieved 19.1% for the gravure-printed flexible PSCs using tBuOH; they also achieved 23.5% PCE for glass-based spin coated PSCs. Application of tert-butyl alcohol: ethyl acetate (tBuOH:EA) can extract DMF/DMSO without dispersing formamidinium lead iodide (FAPbI_3_) or dissolving FAI. The strong coordination of tBuOH:EA makes this possible (Kim et al., 2020). Antisolvent treatment has also been very fruitful in achieving >20% external quantum efficiencies in the perovskite light-emitting diodes, Lin et al. fabricated the visible light-emitting perovskite LED, with EQE 20.3%; in his work they utilized toluene as the antisolvent for fabricating the high-performance perovskite LEDs (Lin et al., 2018).

**FIGURE 6:**
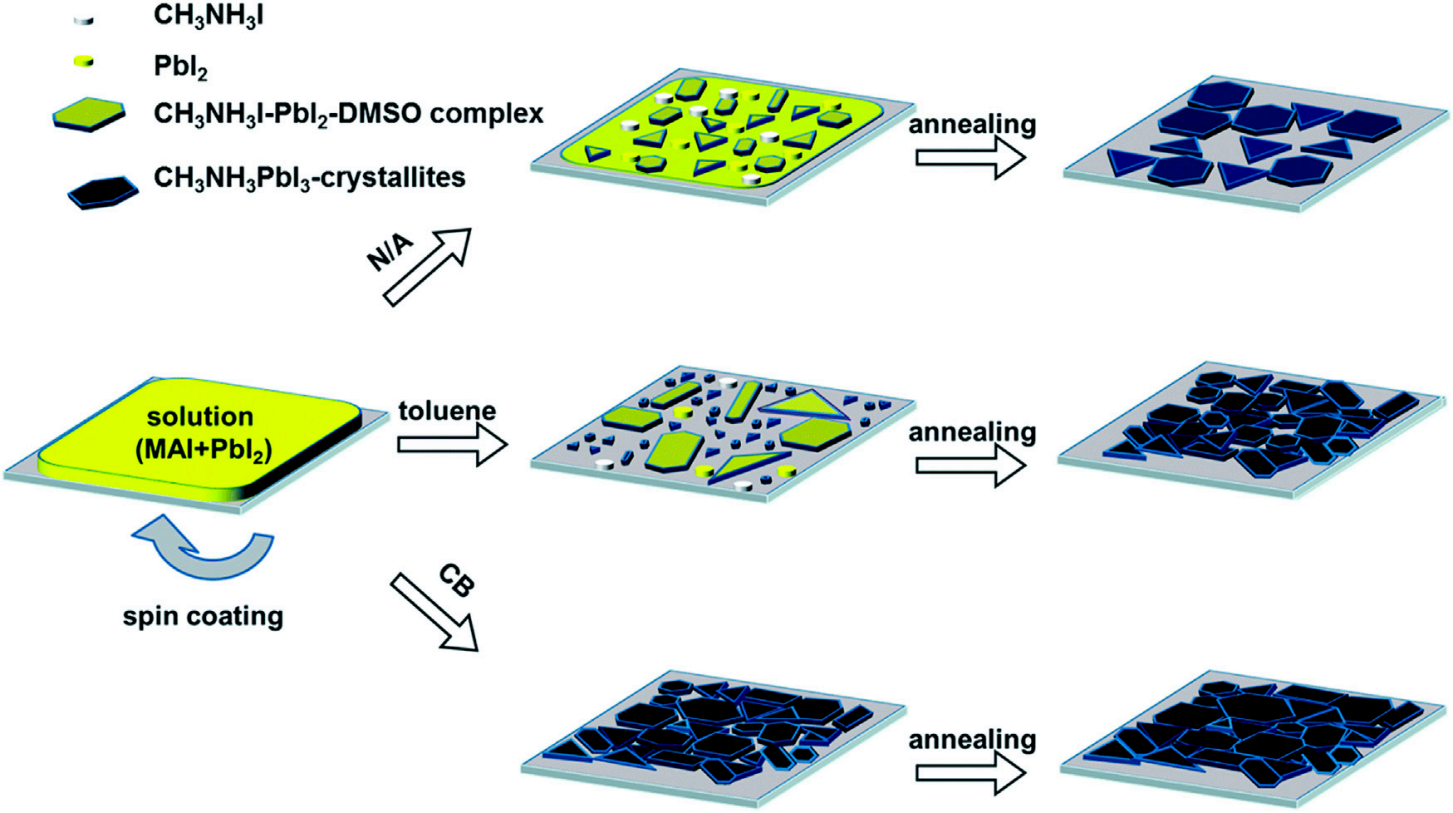
Schematic illustration of the different morphological development with different solvent washing processes. Reproduced with permission (Jun et al., 2014). Copyright 2014, The Royal Society of Chemistry.

In a comparative study of different antisolvents, Jung et al. found that a nonpolar antisolvent such as chlorobenzene (CB) or o-dichlorobenzene (o-CB) with a larger dipole moment, as compared to toluene, can induce the liquid to a crystalline phase transformation instantaneously *via* bypassing the intermediate phase (CH3NH_3_I–PbI_2_–DMSO) (Figure 6) (Jung et al., 2014). The intermediate phase is generally observed when toluene is used as an antisolvent. Using toluene as an antisolvent leads to produce larger grain size as compared to the grain size obtained by the antisolvents such as CB or o-CB. Therefore, using antisolvents with larger dipole moments can result in small grain-sized films with compact, pinhole-free perovskite film morphology suitable for fabrication of the perovskite LEDs; on the other hand, the antisolvents with a low dipole moment can be utilized to produce large grain-sized perovskite films suitable for solar cell application.

## Crystallization Control by Solvent Engineering

### Co-Solvent Method

**FIGURE 7:**
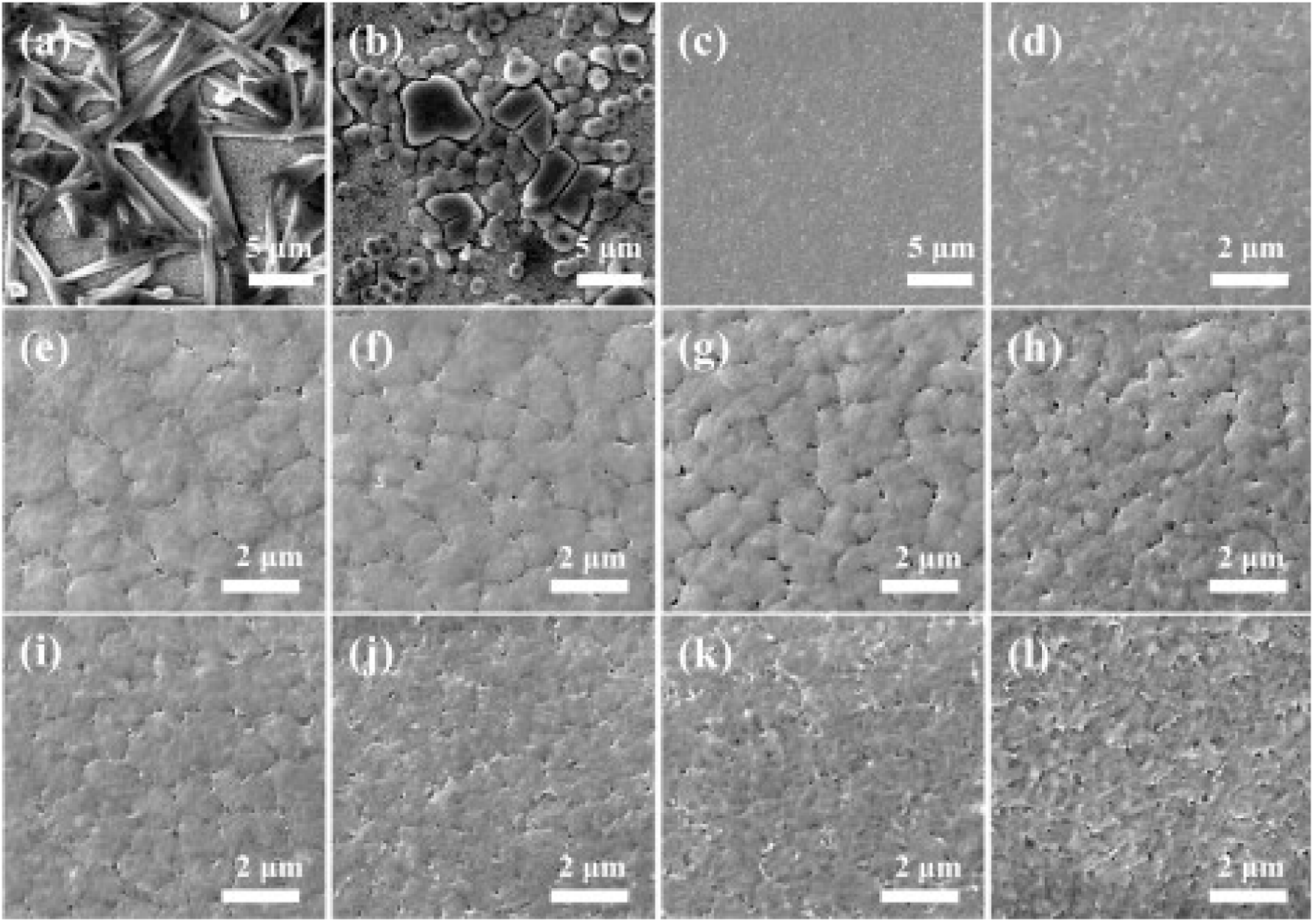
SEM images of MAPbI3 films spin-coated using different solvents. **(A)** Pure DMF, **(B)** Pure GBL, **(C)** and **(D)** Pure DMSO, **(E–H)** DMF–DMSO mixtures containing DMF volume fractions of 20%, 40%, 60%, and 80%, respectively, **(I–L)** GBL-DMSO mixtures containing GBL volume fractions of 20%, 40%, 60%, and 80%, respectively. Reproduced with permission (Cai et al., 2015). Copyright 2015, Elsevier.

DMF (Dimethylformamide), DMSO (Dimethylsulfoxide), DMA (N,N-dimethylacetamide), GBL (γ-butyrolactone), and NMP (N-methyl-2-pyrrolidone) are the most commonly used solvents in making good quality perovskite films for the high-performance perovskite solar cells and light-emitting diode. All of them are aprotic, high boiling point, and low vapor pressure solvents, and they are only useful in making full coverage, pinhole-free compact film when used along with some additional treatments such as antisolvent treatment or gas-assisted crystallization (Lee and Park, 2021). The choice of a suitable aprotic solvent is critically important for preparing the high-quality perovskite films since the interaction between the solvent and the precursors have a strong influence on the crystallization rate and film morphology (Lee and Park, 2021). Sometimes a solvent may not show good solubility for all the perovskite precursors; therefore recently, most of the high-quality perovskite films and high-performance perovskite optoelectronic devices have been fabricated using cosolvents (the binary solvent system). Researchers have developed various techniques to obtain high-quality perovskite films, and formation of intermediate abduct (IA) phase is one of the extremely effective methods in preparing the highly uniform and dense perovskite films. The stability of the IAs is determined by the coordination ability of the solvent molecules with PbI_2_. The role of IA formation is investigated in MASnI_3_-based perovskite films, and it has been observed that by using DMSO (strongly coordinating solvent) as the solvent forms more stable IAs as compared to DMF. Since DMF has a lower coordination affinity, lower boiling point, and higher vapor pressure as compared to DMSO, using DMF in the perovskite film preparation immediately forms the perovskite structure resulting in a black-colored film, whereas in case of DMSO, a gradual yellow to black color change occurs while annealing the films, which indicates that DMSO shows a higher stability of IAs as compared to IAs formed by DMF solvent. Formation of the new IAs from different solvents (PbI_2_•DMSO from DMSO, MAI•PbI_2_•DMF from DMF, MAI•PbI_2_•GBL from GBL, and MAI•PbI_2_•NMP from NMP) has been confirmed, using the XRD and FTIR measurements. The formation of IAs is helpful in controlling the crystal size, crystallization rate, and ultimately the morphology of the perovskite films. Stable IA formation leads to a much compact, uniform, and large grain-sized perovskite films. Cai et al. investigated the effect of the solvent ratio in a binary solvent system. They found that 20–40% of DMF or GBL mixture in DMSO results in an optimum morphology (Figure 7) (Cai et al., 2015). In this method, DMSO plays a dual role of a solvent and also that of a coordinating agent to form a stable intermediate abduct, whereas DMF and/or GBL acts as solvent only. As a result, for the optimized perovskite, Fang et al. tested the performance of dimethylacetamide (DMAc)/N-Methyl-2-pyrrolidone (NMP) cosolvent-based perovskite solar cells and DMAc/DMSO cosolvent-based devices. The PCE of devices fabricated by the DMAc/NMP-based cosolvent was found to be 17.38%, which is of 10% improved efficiency as compared to the devices fabricated by DMAc/DMSO cosolvents (Fang et al., 2017). They also found that the devices fabricated by the DMAc/NMP cosolvent system reached the PCE of 17.09% without the need of thermal annealing, thus indicating that a proper selection of the cosolvent system can lead to device fabrication at low temperatures as low as room temperature.

### Solvent Additive Method

Solvent additives can be used to optimize the surface energy of the solvent, and thus, according to Eq 4, the role of the solvent additives is to control the critical Gibbs free energy of nucleation and critical nuclei radius. Giuliano et al. demonstrated that the addition of α-terpineol into the perovskite precursor solution can improve the crystallinity, uniformity, and photovoltaic performance (Giuliano et al., 2021). In another study, Zhang et al. studied several classes of solvents to improve the interfacial contact between the active perovskite layer and nonwetting HTL layer. In this study, on the effect of solvent additive, four different types of solvent additives were added into the perovskite precursor solution. They found that the contact angles for ethyl acetate (EA), chlorobenzene (CB), 2-butanol, and H_2_O-added solutions showed the contact angles (with precursor) of 19.2°, 22.4°, 21.6°, and 47.4°, respectively. The surface coverage for the controlled samples (without any solvent additive) was found to be around 20–30% only. Surface coverage for EA and CB additives was improved to more than 95% (Zhang et al., 2021). They found a positive correlation between the contact angle and grain size obtained by adding various solvent additives. Although H_2_O as a solvent additive yielded large grain sizes suitable for the solar cell application, however, poor film coverage remains a challenge. Therefore, they explored the effect of H_2_O + CB as a mixed additive to combine the advantage of excellent film coverage along with the larger grain size. By optimizing the ratio of CB and H_2_O, they achieved the champion cell efficiency of 22.1% with a mixed-cation perovskite (FA_0.83_Cs_0.07_MA_0.13_PbI_2.64_Br_0.39_) stoichiometry.

## Conclusion

In summary, we have reviewed the latest, advanced strategies to control the perovskite crystal growth in fabricating the high-quality hybrid halide perovskite thin films, suitable for optoelectronic devices. It has been found that the key parameter deciding the nucleation and subsequent growth of the nucleated particles is the Gibbs free energy. We mainly focused on the three most popular strategies, namely, substrate temperature treatment, antisolvent treatment, and cosolvent engineering. We found that these techniques are found to be highly effective in controlling the film morphology and obtaining the high-performance perovskite solar cells and perovskite light-emitting diodes. The condition for the nucleus formation is to achieve the supersaturation of the perovskite precursor solution, either by heat treatment or *via* solvent extraction by an antisolvent treatment. Preheating the substrate can affect surface-induced nucleation and control the crystal growth. It has been observed that the formation of uniform pinhole-free perovskite films can be realized by elevating the substrate temperature close to the equilibrium melting point (T_m_). However, only those substrate temperatures below T_m_ are thermodynamically favorable. The temperature-dependent growth rate reveals that the growth of the nucleated particles is maximum when the substrate temperature is kept closer to T_m_. The chemical potential of the nucleic site can be tuned in order to tune between the FM and VW growth mode with the help of tuning the delay of the antisolvent treatment. This helped us to tune the perovskite film morphology for the application in the high-performance devices. Also, the rate of antisolvent dripping plays a significant role in improving the crystallization process; a slow rate of antisolvent dripping is found to result in better quality perovskite films and solar cells. Apart from this, the solvent boiling point, coordination affinity, and dipole strength also play a significant role in deciding the ultimate film morphology, defect density, and charge transfer resistance. Solvents with a higher coordination affinity such as DMSO help to slow down the crystal growth rate and achieve the larger sized grains suitable for the perovskite solar cells. An optimized ratio of the low and high coordination affinity solvent can help to control the grain size and film morphology in accordance with the application.
